# Quinine Blindness

**Published:** 1913-12

**Authors:** 


QUININE BLINDNESS. Zani, *Riv. Veneta di Sci. Med.,* August 15, 1911, reports a case of a woman who attempted suicide by swallowing 20 grams in tablets. Toxic symptoms began in five hours. Gastric lavage, coffee, purgatives and enemata, sinapisms and hot applications prevented death. Visual hallucinations, mydriasis, retinal oedema with blindness, and tinnitus aurium were noted. Central vision returned in fifteen days, peripheral vision and color vision, more gradually. The disc was atrophic with small vessels.

